# Graph learning for particle accelerator operations

**DOI:** 10.3389/fdata.2024.1366469

**Published:** 2024-04-11

**Authors:** Song Wang, Chris Tennant, Daniel Moser, Theo Larrieu, Jundong Li

**Affiliations:** ^1^Department of Electrical and Computer Engineering, University of Virginia, Charlottesville, VA, United States; ^2^Thomas Jefferson National Accelerator Facility, Newport News, VA, United States

**Keywords:** Graph Neural Network, particle accelerator, self-supervised learning (SSL), supervised training, graph learning algorithm

## Abstract

Particle accelerators play a crucial role in scientific research, enabling the study of fundamental physics and materials science, as well as having important medical applications. This study proposes a novel graph learning approach to classify operational beamline configurations as good or bad. By considering the relationships among beamline elements, we transform data from components into a heterogeneous graph. We propose to learn from historical, unlabeled data via our self-supervised training strategy along with fine-tuning on a smaller, labeled dataset. Additionally, we extract a low-dimensional representation from each configuration that can be visualized in two dimensions. Leveraging our ability for classification, we map out regions of the low-dimensional latent space characterized by good and bad configurations, which in turn can provide valuable feedback to operators. This research demonstrates a paradigm shift in how complex, many-dimensional data from beamlines can be analyzed and leveraged for accelerator operations.

## 1 Introduction

A particle accelerator is a scientific device used to accelerate charged particles, such as protons or electrons, to very high speeds and energies. These accelerators are used in various fields of research, including particle physics, nuclear physics, materials science, medicine, and for industrial applications. Particle accelerators play an instrumental role in advancing our understanding of the fundamental properties of matter and the universe. In addition to particle physics, accelerators also have applications in other fields. They are used in nuclear physics to investigate the structure and forces in the nucleus, in materials science to analyze the structure and properties of materials, in medicine for diagnostics and treatment, radiation therapy, and medical imaging (Brüning and Myers, 2016).

Particle accelerators represent some of the most complex scientific instruments ever designed, built, and operated. To guide operators, enormous efforts are expended to create high-fidelity simulations of accelerator beamlines. While these simulations provide an initial starting point for operators, there exists a gap between the ideal simulated entity and the real-world implementation. Bridging that gap requires a time-consuming task known as beam tuning. Beam tuning is an iterative process that is often slow to converge, either because it requires interaction with a simulation or by looking at a limited number of diagnostics. For many accelerator facilities, beam tuning represents one of the dominant sources of machine downtime. Our work proposes a data-driven framework in which beamline configurations are mapped to a low-dimensional space, regions of good and bad configurations are identified, and subsequent beamline configurations are classified accordingly. Such a tool would be a valuable addition in the control room for operators.

**Figure 1:**
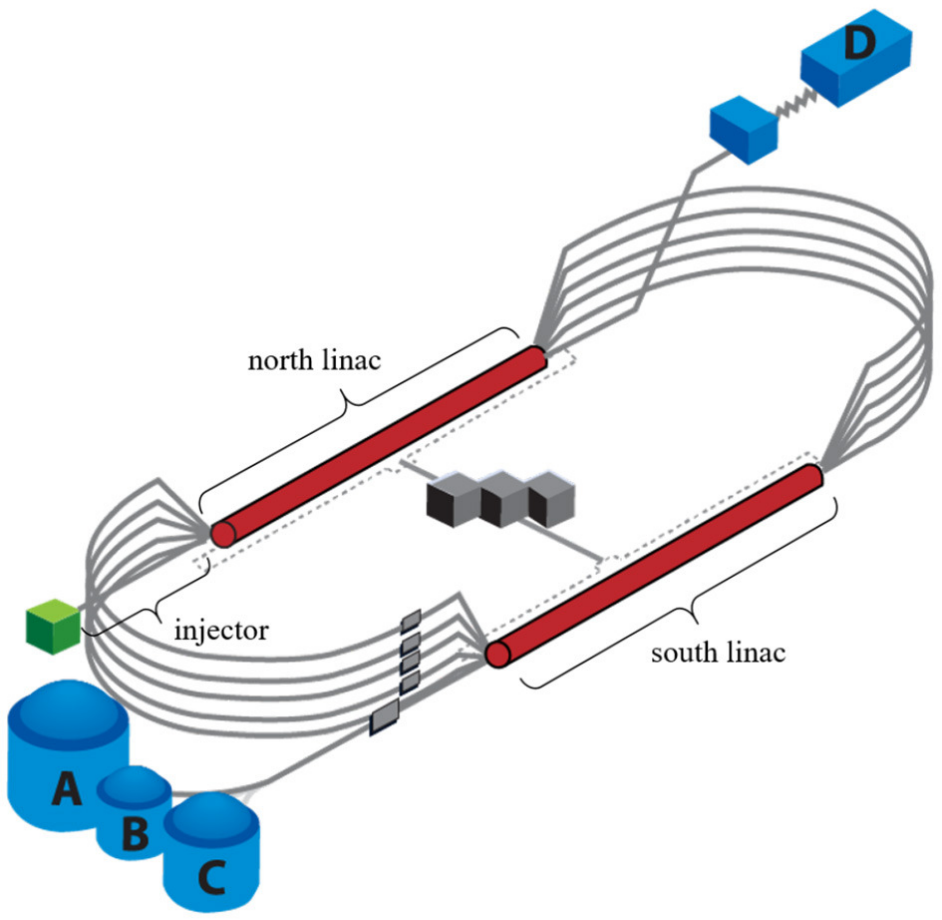
Schematic of the CEBAF accelerator. Electrons are generated in the injector. Multiple passes through the north and south linacs accelerate beam to multi-GeV energies. Beam is then sent to the four nuclear physics experimental halls (A, B, C, and D).

The Continuous Electron Beam Accelerator Facility (CEBAF) at the Thomas Jefferson National Accelerator Facility is a high power, recirculating linac capable of delivering electron beams to four different experimental nuclear physics end stations simultaneously (Reece, 2016). Figure 1 shows a schematic of CEBAF. For the purpose of this work, we focus on the CEBAF injector beamline. The injector is well suited due to its manageable size, diversity of beamline components, and abundance of tuning data reflected in the operational archiver. More specifically, because the formation and evolution of the beam at low energy are critical to performance, the injector represents a region with a lot of operational activity. This translates to a wealth of historical data that can be used for training a model.

In this work, we propose GLOPA (Graph Learning for Operations of Particle Accelerators), a novel data-driven approach for classifying a beamline configuration by leveraging deep learning over structured data, i.e., graphs. Specifically, we propose to represent a beamline configuration at any arbitrary date and time as a heterogeneous graph and design a novel Graph Neural Network (GNN) (Kipf and Welling, 2017; Veličković et al., 2018; Ying et al., 2018; Wu et al., 2019; Xu et al., 2019; Han et al., 2022) framework to extract low-dimensional representations that can be visualized in two-dimensions (Ultsch, 2003; Van der Maaten and Hinton, 2008; Barshan et al., 2011). Furthermore, by embedding months of historical operational data, and using self-supervised learning (Hassani and Khasahmadi, 2020; Gao et al., 2021; Suresh et al., 2021; Wan et al., 2021) together with supervised learning (Li et al., 2018; Khosla et al., 2020; Jin et al., 2021; Akkas and Azad, 2022), regions of parameter space characterized by good and bad configurations can be mapped out. In the future, such a visualization could be used in real-time to aid beam tuning by providing human operators with immediate visual feedback about whether changes they make are moving the system in the right direction, that is, toward or away from a good region of parameter space. The main contributions of this paper are as follows: (1) **Formulation**. Our work is the first to represent accelerator beamlines as heterogeneous and directed graphs for deep learning. (2) **Algorithm**. We propose a novel framework with self-supervised and supervised strategies to learn the latent beamline embeddings from both labeled and unlabeled data. (3) **Evaluation**. The proposed framework is evaluated on several real-world beamline datasets. Promising performance verifies the effectiveness of our framework for beamline classification and for identifying good and bad regions of a low-dimensional, latent space.

## 2 Preliminaries

### 2.1 Data preparation

**Figure 2:**
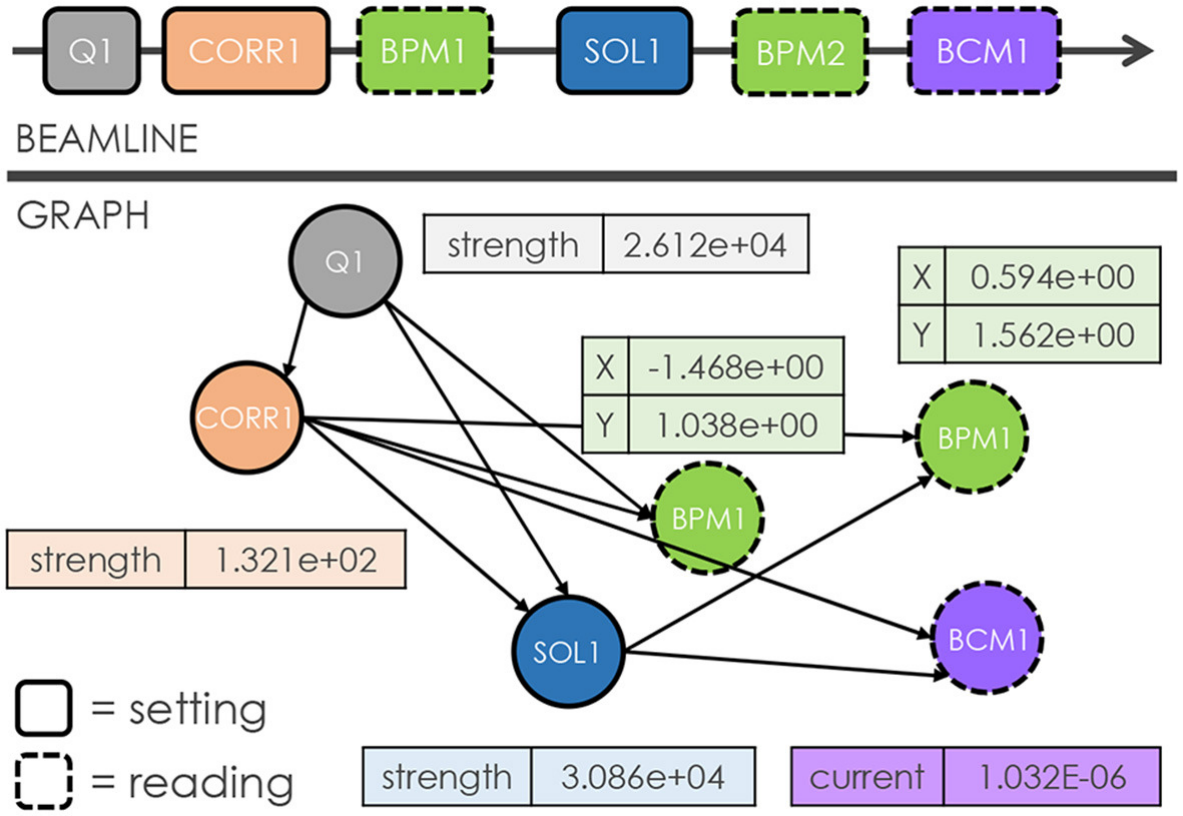
Illustrations that showcase an arbitrary accelerator beamline **(top)** and our approach for constructing a corresponding graph **(bottom)**. Here, each node represents an individual element, while the node features correspond to the relevant parameters of the respective element. The edges between nodes are determined by a user-defined window size of 2. These edges are directed to reflect the fact that an element cannot impact upstream elements in the beamline.

To illustrate the concept of representing a beamline as a graph, consider the following example in Figure 2. The beamline consists of different types of elements: beam current monitors (BCMs), beam position monitors (BPMs), quadrupoles, solenoids, and correctors, which are all represented as nodes. Specifically, each node type consists of several unique features. For example, quadrupoles, correctors, and solenoids carry a single value that indicates their field strength, a BCM reports its beam current value, and a BPM contains two features for horizontal and vertical beam positions, respectively. In this manner, the resulting graph is a heterogeneous, directed graph, where heterogeneity originates from nodes of different types and directionality arises from the edges. The edges between nodes are determined by a user-defined “window” concept. In this case, a user-defined window size of 2 is used, which means each ***setting*** node is connected to the two immediate downstream setting nodes, including any ***reading*** nodes in between. Setting nodes correspond to those elements that human operators can modify during routine beam tuning tasks (e.g., quadrupole, solenoid, and corrector), while reading nodes represent diagnostics that passively read back data (e.g., BPM and BCM). Additionally, the directed edges reflect the non-recirculating nature of our beamline topology, where an element (i.e., node) cannot influence anything upstream. It should be noted that the window size is controllable, which means different graph representations may benefit from varying window sizes, depending on the specific downstream tasks and beamline characteristics.

Moreover, utilizing a graph framework naturally enables the inclusion of global beamline parameters. Specifically, in our case, a master node is connected to all other nodes and thus can incorporate readings from global values, such as beam current, temperature readings in the beamline enclosure, outdoor temperature and humidity, date and time information, or even electronic log entries.

For this work, a single beamline graph is comprised of 12 distinct node types, 207 total nodes, 393 total node features, and 528 edges (for a window size of 2), where a master node connects to all other nodes. Note that each node type contains specific node features, which can be significantly different in magnitude, due to the different physical features they quantify. Therefore, we propose to perform element-wise normalization for each node. Specifically, within each dataset, we obtain the mean and standard deviation of each individual node and then standardize the features of each.

For this work, we collect four datasets which are summarized in Table 1. For the TRAIN dataset, we collect data between October 1, 2021 and February 8, 2022 and construct graphs at 20-min intervals. This dataset will be used for self-supervised training. We obtain the GOOD and BAD datasets from expert-annotated data, using a fraction of them for supervised training and the remainder as test data (see Appendix A). For the JAN-2022 dataset, we collect data from beam operations in January 2022 at one-hour intervals, which will be used to evaluate our framework.

**Table 1:** Statistics of four beamline datasets.

**Dataset**	**Time interval**	**# Graphs**	**# Edges**	**# Nodes**	**# Types**
TRAIN	20 min	5,827	528	207	12
GOOD	Inconsistent	354		
BAD	Inconsistent	254		
JAN-2022	1 h	353		

Note that GOOD and BAD datasets are labeled, while the others are unlabeled.

### 2.2 Problem formulation

In this subsection, we formally define the problem of beamline analysis. A given dataset *D* consists of a specific number of graphs, i.e., *D* = {*G*_1_, *G*_2_, …, *G*_|*D*|_}. Here each graph *G* is represented by *G* = (V, E, **X**) with its label *y* ∈ Y, where Y is the total class set in this dataset. Specifically, V and E represent the node set and edge set, respectively. Each graph *G* can also be denoted by an adjacency matrix **A** ∈ ℝ^*n*×*n*^, where *n* is the number of nodes in *G*. Here **A**_*i, j*_ is the intersection of the *i*-th row and the *j*-th column of **A**. Moreover, **A**_*i, j*_ = 1 if the *i*-th node connects to the *j*-th node, and **A**_*i, j*_ = 0, otherwise. Since beamline graphs are heterogeneous, we further introduce a node type mapping function τ:V→T. Here T denotes the node type set. In this manner, we can represent the node type of the *i*-th node *v*_*i*_ as τ(*i*). Then the node feature set can be represented as X = {**x**_1_, **x**_2_, …, **x**_*n*_}, where ${\text{x}}_{i}\in {\mathbb{R}}^{d_{\tau \left(i\right)}}$ denotes the node attributes of *v*_*i*_, and *d*_τ(*i*)_ is the corresponding dimension size of type τ(*i*).

Our objective is to determine whether a beamline at a specific date and time (represented as a graph) represents a good or bad configuration. This task can be formulated as a binary classification problem, where the class set is defined as Y = {0, 1}. In general, we aim at learning from labeled beamline graphs and predicting the label for a given beamline graph from the class set Y. However, it is important to note that beamline graphs display heterogeneity due to the presence of different node types. Consequently, directly applying existing Graph Neural Networks (GNNs) is infeasible. In order to handle the heterogeneity, we must consider the specific properties associated with different node types.

## 3 Methodology

### 3.1 Latent embedding

**Figure 3:**
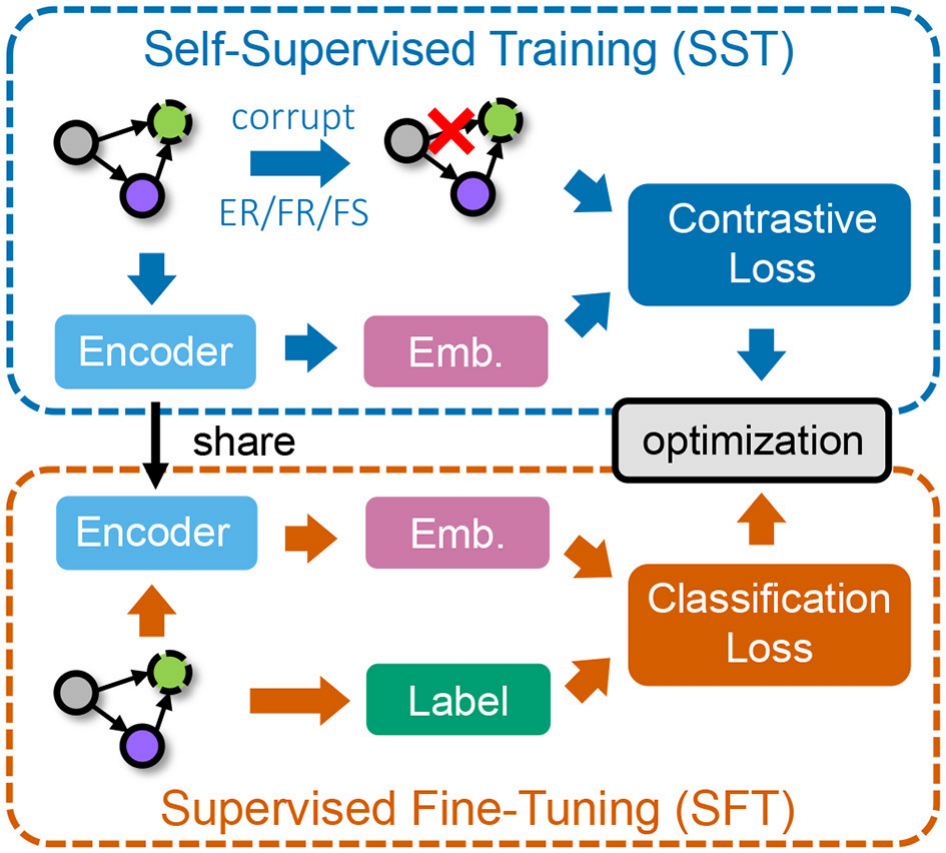
The overall workflow of our framework. We first conduct self-supervised training (SST) on unlabeled data via contrastive learning **(top)** and then perform supervised fine-tuning (SFT) on a smaller set of labeled data **(bottom)**.

In this section, we introduce our approach for learning graph embeddings of CEBAF injector beamline graphs based on self-supervised and supervised learning, while considering the heterogeneity of different node types. The framework is illustrated in Figure 3.

#### 3.1.1 Heterogeneous graph convolution

Inspired by HGAT (Hu et al., 2019), we propose encoding beamline graphs based on a heterogeneous graph convolution, which handles variations in features from different node types by projecting them into an implicit common space via linear layers. Formally, consider a beamline graph *G* = (V, E), where V and E represent the set of nodes and edges, respectively. For graph *G*, we introduce its self-connected adjacency matrix as **A**′ = **A**+**I**, where **I** is the identity matrix of size *n*, and *n* = |V|. We further denote *M* as the degree matrix, i.e., ${\text{M}}_{ii}=\overset{n}{\underset{j=1}{\sum }}{\text{A}}_{i,j}$.

We first start with the convolution process operation in GCN (Kipf and Welling, 2017) on homogeneous graphs. The layer-wise propagation rule can be represented as follows:


(1) $$H^{\left(l+1\right)}=\text{ReLU}\left(\tilde{A}\cdot \left(H^{\left(l\right)}\cdot \;W^{\left(l\right)}+b^{\left(l\right)}\right)\right),$$

where $\tilde{\text{A}}={\text{A}}^{\prime }{\text{M}}^{-1}$ represents the normalized adjacency matrix. $W^{\left(l\right)}\in {\mathbb{R}}^{d^{\left(l\right)}\times d^{\left(l+1\right)}}$ and $b^{\left(l\right)}\in {\mathbb{R}}^{d^{\left(l+1\right)}}$ denote the layer-specific weight parameters of a linear layer. **H**^(*l*)^ denotes hidden representations of nodes in the *l*-th layer of the GCN, with a dimension size of *d*^(*l*)^. ReLU(·) is an activation function, which sets negative entries in the input as zero. Moreover, in Equation (1), the node features are used as the input for the first layer of GCN, i.e., **H**^(0)^ = **X**, where **X** is the node feature matrix.

However, applying the traditional GCN operation directly to beamline graphs is not feasible due to the heterogeneity of node types and varying node feature sizes. To overcome this challenge, we propose to utilize heterogeneous graph convolution (Chang et al., 2015; Hu et al., 2019, 2020; Liu et al., 2020), which enables the incorporation of information from different node types. This is achieved by projecting the node attributes of different types to an implicit common space:


(2) $${\tilde{X}}_{\tau }=X_{\tau }\cdot W_{\tau }+\;b_{\tau },\;\tau \in T,$$

where ${\text{W}}_{\tau }\in {\mathbb{R}}^{d_{\tau }\times d^{\left(0\right)}}$ and ${\text{b}}_{\tau }\in {\mathbb{R}}^{d^{\left(0\right)}}$ are the weight parameters of projection for node type τ. *d*_τ_ is the dimension size of node attributes for node type τ. T = {τ_1_, τ_2_, …, τ_*t*_} is the set of node types in beamline graphs, and *t* = |T| is the number of node types. In this manner, we can perform heterogeneous graph convolution as follows, based on Equation (2):


(3) $$H^{\left(l+1\right)}=\text{ReLU}\left(\sum_{\tau \in T}{\tilde{A}}_{\tau }\cdot \left(H_{\tau }^{\left(l\right)}\cdot \;W_{\tau }^{\left(l\right)}+b_{\tau }^{\left(l\right)}\right)\right),$$

where ${\text{W}}_{\tau }^{\left(l\right)}\in {\mathbb{R}}^{d^{\left(l\right)}\times d^{\left(l+1\right)}}$ and ${\text{b}}_{\tau }^{\left(l\right)}\in {\mathbb{R}}^{d^{\left(l\right)}}$ are the layer-specific weight parameters of a linear layer for node type τ. Note that we employ different weight parameters for various node types to tackle the heterogeneity problem by projecting them into an implicit common space ${\mathbb{R}}^{d^{\left(l+1\right)}}$. Moreover, ${\tilde{\text{A}}}_{\tau }\in {\mathbb{R}}^{n\times n_{\tau }}$ is a submatrix of $\tilde{\text{A}}$, where the rows represent all the nodes in *G* while columns represent their corresponding neighboring nodes with node type τ. Here *n* = |V| and *n*_τ_ = |V_τ_|, where V_τ_ denotes the set of nodes in *G* with node type τ. In this manner, the (*l*+1)-th layer node representations **H**^(*l*+1)^ are obtained by aggregating information from the previous layer node representations of their neighboring nodes, i.e., ${\text{H}}_{\tau }^{\left(l\right)}$, with different node types τ ∈ T.

#### 3.1.2 Heterogeneous attention mechanism

Due to the inherent heterogeneity in the beamline graphs, edges connecting different nodes can possess varying importance for each node. For instance, a setting node may have multiple neighboring nodes, but will exert a greater influence on those nodes that correspond to being in closer proximity on the physical beamline.

To effectively leverage such information, we propose a novel heterogeneous attention mechanism that captures the diverse importance at both the node-level and type-level. This mechanism enables us to consider the varying degrees of impact that different nodes and node types exhibit in the beamline analysis:


(4) $$b_{ij}=\delta_{ik}\cdot \sigma (v_{\tau (i)}\circ \;h_{i})\cdot \sigma (v_{\tau (j)}\circ \;h_{j}),$$

where **v**_τ(*i*)_ is the attention vector for the node type of *v*_*i*_, i.e., τ(*i*). δ_*ij*_ = 1 if node *v*_*i*_ connects to node *v*_*j*_, and δ_*ij*_ = 0, otherwise. ◦ denotes the element-wise multiplication operation. σ(·) is the Sigmoid function with σ(*x*) = 1/(1+exp(−*x*)). After normalization with ${\tilde{b}}_{ij}=b_{ij}/\overset{n}{\underset{k=1}{\sum }}b_{ik}$, we can replace the adjacency matrix in Equation (3) with the obtained attention value ${\tilde{b}}_{ij}$ from Equation (4).

### 3.2 Self-supervised training

To effectively leverage unlabeled historical beamline data, we devise an innovative Self-Supervised Training (SST) process. Specifically, we propose to leverage the concept of graph contrastive learning (Hassani and Khasahmadi, 2020; Jin et al., 2021; Zhu et al., 2021) due to its effectiveness in learning expressive node and graph representations. In particular, graph contrastive learning aims to maximize consistency between differently augmented views of the original graph, while distinguishing the original graph and another graph (Oord et al., 2018; Qiu et al., 2020; You et al., 2020). However, in beamline analysis, our ultimate goal is to identify the class of graphs, instead of classifying different nodes. Therefore, we propose a graph contrastive learning strategy, which considers the consistency between graph and node representations. Specifically, we propose to maximize the similarity between the learned graph representation and any node representations, while minimizing the similarity between the graph representation and other corrupted node representations (Velickovic et al., 2019). We can formally express the self-supervised training loss based on graph contrastive learning as follows:


(5) ℒSST=−1n∑i=1nlog s(hi,h*)−1n∑i=1nlog (1−s(h˜i, h*)),

where ${\text{h}}_{i}={\text{h}}_{i}^{\left(L\right)}\in {\mathbb{R}}^{d^{\left(L\right)}}$ is the learned representation of the *i*-th node *v*_*i*_ in *G* after processed by an *L*-layer GNN in our framework. Correspondingly, ${\tilde{\text{h}}}_{i}$ is the representation of $\tilde{v_{i}}$ in $\tilde{G}$, which is a corrupted view of *G* and will be introduced later. $s\left({\text{h}}_{i},{\text{h}}^{*}\right)=\sigma \left({\text{h}}_{i}\cdot {\text{h}}^{*}\right)$ measures the similarity between **h**_*i*_ and h^*^. Moreover, h^*^ is the learned graph representation of *G* via the following process:


(6) $$h^{*}=\sigma \left(\frac{1}{n}\sum_{i=1}^{n}\left(h_{i}\cdot h_{m})h_{i}/(\sum_{j=1}^{n}h_{j}\cdot h_{m}\right)\right).$$

Note that here we employ an attention mechanism that considers the varying importance of different nodes to obtain h^*^. Since the master node connects to all nodes in *G* and maintains global information, we consider its impact on all nodes as the importance. Specifically, we learn the weight of **h**_*i*_ via the similarity between **h**_*i*_ and **h**_*m*_ (i.e., the representation of the master node in *G*).

To obtain the corrupted view $\tilde{G}$ of *G*, we propose three strategies while considering the heterogeneity in beamline graphs.

- **Edge removing (ER)**. In this strategy, we randomly remove edges in the graph according to a pre-defined removal rate.
- **Feature removing (FR)**. In this strategy, we randomly set node feature values to zero for nodes in the graph, based on a pre-defined removal rate.
- **Feature shuffling (FS)**. In this strategy, for each node type, we randomly select a specific ratio of nodes and then randomly swap their attributes. Note that the shuffling is performed within each node type, as the number of features may vary with the type.

By applying these strategies, we can achieve corrupted graphs $\tilde{G}$ for our self-supervised training, based on Equation (5).

### 3.3 Supervised fine-tuning

In this subsection, we provide details of the Supervised Fine-Tuning (SFT) process in our framework. Although we have managed to learn from the complex structural information in beamline graphs through our Self-Supervised Training (SST), it still remains challenging to effectively utilize the limited number of labeled examples for training. Therefore, it is crucial to develop a supervised fine-tuning strategy that can improve performance with a small labeled dataset.

In particular, our objective in this process is to leverage the supervision information from annotated beamline graphs. Since beamline analysis aims to distinguish good and bad beamline configurations, we leverage the binary cross-entropy loss to train our framework. Specifically, the supervised fine-tuning loss can be formulated as:


(7) $${ℒ}_{SFT}=-\frac{1}{\left|D\right|}\sum_{i=1}^{\left|D\right|}y_{i}\cdot \log p_{i}-\frac{1}{\left|D\right|}\sum_{i=1}^{\left|D\right|}\left(1-y_{i}\right)\cdot \log(1-p_{i}),$$

where *D* is the given dataset with *D* = {*G*_1_, *G*_2_, …, *G*_|*D*|_}. *y*_*i*_ ∈ {0, 1} is the label of *G*_*i*_, and *p*_*i*_ ∈ ℝ is the output probability of *G*_*i*_, obtained by $p_{i}=\sigma \left({\text{h}}_{i}^{*}\cdot {\text{w}}^{*}+b^{*}\right)$. Here ${\text{h}}_{i}^{*}\in {\mathbb{R}}^{d^{\left(L\right)}}$ is the learned graph representation of *G*_*i*_, based on Equation (6). w^*^ ∈ ${\mathbb{R}}^{d^{\left(L\right)}}$ and b^*^ ∈ ℝ are learnable weight parameters of a linear layer. In this way, we can obtain the output probability *p*_*i*_ for beamline analysis on *G*_*i*_ via Equation (7).

## 4 Experiments

To achieve an empirical evaluation of our proposed framework GLOPA, we conduct experiments on real beamline datasets collected from the CEBAF injector.

### 4.1 Experimental setup

Our overall framework is implemented based on PyTorch (Paszke et al., 2017), scikit-learn (Pedregosa et al., 2011), and PyTorch Geometric (Fey and Lenssen, 2019). We run the model on a single 48GB NVIDIA A6000 GPU with a batch size of 16 for self-supervised training (SST) and 32 for supervised fine-tuning (SFT). The model is trained for a total of 200 epochs with a learning rate of 0.001. The hidden size of GNN models in our framework is set to 16, and the number of layers is set to 3. We optimize the model based on Adam (Kingma and Ba, 2015). The ratio used in ER/FR/FS is set to 0.3. For all the baselines and the proposed framework, we repeat each run ten times to obtain the averaged scores. For SST in our framework, we utilize unlabeled data in the TRAIN dataset for model training. In each run, we randomly leverage 80% of data in GOOD and BAD datasets for SFT and 10% for validation, with the other 10% left for model evaluation.

### 4.2 Baselines and evaluation metrics

In our experiments, we compare our framework GLOPA to the following baselines for performance evaluation:

- **MLP (multi-layer perceptron):** In this baseline, we ignore the structural information in beamline configurations and represent each of them as a concatenated feature vector. The feature is input into a fully-connected layer for classification.
- **1D-CNN (1D-convolutional neural network) (Kiranyaz et al., 2021):** In this baseline, we use a one-dimensional CNN model to encode the concatenated feature vector, which can capture the correlations across different dimensions.
- **GCN (Kipf and Welling, 2017):** In this baseline, we employ the vanilla GCN to learn latent embeddings for beamline graphs. To adapt GCN to heterogeneous graphs, we construct a new feature space by concatenating the feature spaces of different node types.
- **GLOPA\SST:** In this variant, we remove the SST component such that only SFT is performed, which means we do not utilize the unlabeled data and only rely on labeled data.

It is worth noting that in our scenario, we aim to identify beamline configurations as good or bad. Thus, we formulate the task of beamline analysis as binary graph classification.

### 4.3 Comparative results

In this subsection, we compare the performance of our GLOPA framework with other baselines for the task of beamline classification. We provide the results in Table 2. From the results, we can make the following observations:

- GLOPA consistently outperforms other baselines in terms of Accuracy (ACC), Area Under Curve (AUC), and F1-Score (F1). The results strongly indicate the superiority of our framework for beamline analysis.
- Conventional methods struggle to achieve competitive performance. This can be attributed to their limited ability to effectively capture the structural and heterogeneous information present in beamline graphs.
- Different corruption strategies employed in our SST process exhibit varying performances. Specifically, the strategy of feature shuffling (FS) achieves the best results. This is because such a strategy can benefit from the heterogeneity of beamline graphs by considering the diverse meanings of features across different node types. On the other hand, the edge removing (ER) strategy demonstrates less competitive performance compared to the other two, especially in terms of ACC and F1. This could be due to the fact that the number of edges remains constant in beamline graphs. Therefore, relying solely on edge-related structural information could be less effective.
- The performance of GLOPA without SST remains suboptimal, indicating that SST conducted on a larger number of unlabeled graphs is crucial for beamline analysis.

**Table 2:** The beamline analysis results of our framework and other baselines.

**Method**	**ACC**	**AUC**	**F1**
MLP	85.05 ± 3.75	84.81 ± 3.90	82.83 ± 3.47
1D-CNN	86.35 ± 3.91	85.41 ± 3.78	84.10 ± 1.50
GCN	86.96 ± 3.37	90.07 ± 1.26	87.08 ± 1.07
GLOPA\SST	88.32 ± 1.23	90.05 ± 1.35	89.34 ± 1.01
GLOPA-ER	93.17 ± 1.67	96.02 ± 1.21	93.43 ± 1.79
GLOPA-FR	95.97 ± 1.61	96.57 ± 1.68	96.31 ± 1.98
GLOPA-FS	**96.76**±**1.52**	**98.17**±**1.79**	**96.29**±**1.60**

The best results are shown in **bold**.

### 4.4 Effects of different types of nodes

As introduced in Section 2.1, our graphs contain nodes of different types. The node types can be broadly categorized as setting and reading nodes. Setting nodes represent beamline components whose attributes can be directly modified by human operators during routine beam tuning tasks, whereas reading nodes passively read signals from diagnostics regarding the state of the beamline or the beam itself. Therefore, the information residing in each type of node is inherently different. In this subsection, we explore the effects of different node types and features on the model performance. In particular, we consider the following variants of data for experiments: (1) setting-only (reading-only), which only considers setting (reading) nodes. (2) quadrupole-only, which considers all reading nodes and only setting nodes of type quadrupole. (3) BPM-only, which considers all setting nodes and only reading nodes of type BPM, as BPM nodes are the majority of reading nodes. We use the Feature Shuffling (FS) of GLOPA, as it achieves the best performance. From the results presented in Table 3, we first observe that removing any type of nodes results in a deterioration of the performance, demonstrating that information in all types of nodes is useful. Second, removing setting nodes leads to a larger performance drop, compared to removing reading nodes. This result indicates that the setting nodes contain more important information for predictions, which aligns with physical intuition, as setting nodes have a direct impact on all downstream nodes including reading nodes. Third, preserving more important node types can provide a smaller performance drop, such as specific types of nodes that are the majority of setting or reading nodes. For example, preserving nodes of type quadrupole provides better results than preserving nodes of type corrector, as quadrupole generally have a larger impact on the beam.

**Table 3:** The beamline analysis results of our framework while keeping nodes of specified types.

**Variant**	**ACC**	**AUC**	**F1**
All nodes	**96.76**±**1.52**	**98.17**±**1.79**	**96.29**±**1.60**
Setting-only	95.72 ± 1.35	97.01 ± 1.88	96.19 ± 2.03
Reading-only	86.84 ± 3.25	91.57 ± 2.32	87.73 ± 1.67
Quadrupole + reading	96.71 ± 1.24	96.32 ± 2.31	96.09 ± 1.65
Corrector + reading	89.47 ± 2.13	93.93 ± 1.07	90.18 ± 1.56
Setting + BPM	96.05 ± 1.09	97.23 ± 0.63	95.57 ± 1.87

The best results are shown in **bold**.

### 4.5 Effects of window size

To generate graphs, the edges between nodes are created based on a user-defined window size *w*, which is set as *w* = 2 in our experiments. In particular, for each setting node *v*, we connect it to the *w* immediate downstream setting nodes, including all reading nodes in between. We define such a rule to ensure that the influence of each node on the downstream nodes is captured within the created edges. As the influence of nodes may diminish on downstream nodes further away, we set a window size *w* to restrict the number of edges created. In practice, the window size is a hyperparameter, which requires exhaustive searching to obtain the optimal value. Here we provide experimental results with different window sizes in Table 4, using the Feature Shuffling (FS) variant of GLOPA. From the results, we observe that the best prediction results are for *w* = 2 (better accuracy) or *w* = 3 (slightly higher AUC). Compared to the results of *w* = 1, we can infer that each setting node is able to influence multiple downstream setting and reading nodes and therefore, it is necessary to set a window size larger than *w* = 1. On the other hand, the results of *w* = 5 are less competitive, compared with other variants. These results demonstrate that while each setting node affects a range of other downstream nodes, the range is typically not large. As a result, with a larger window size, the created edges are denser and potentially involve noisy information that is harmful to predictions.

**Table 4:** The beamline analysis results of our framework with different values of *w* (window size).

**Variant**	**ACC**	**AUC**	**F1**
*w* = 1	95.39 ± 1.36	98.13 ± 1.35	95.89 ± 2.03
*w* = 2	**96.76**±**1.52**	98.17 ± 1.79	**96.29**±**1.60**
*w* = 3	94.74 ± 1.71	**98.87**±**1.13**	95.27 ± 1.89
*w* = 5	94.07 ± 2.25	97.57 ± 1.53	94.64 ± 1.79

The best results are shown in **bold**.

### 4.6 Semi-supervised setting

**Figure 4:**
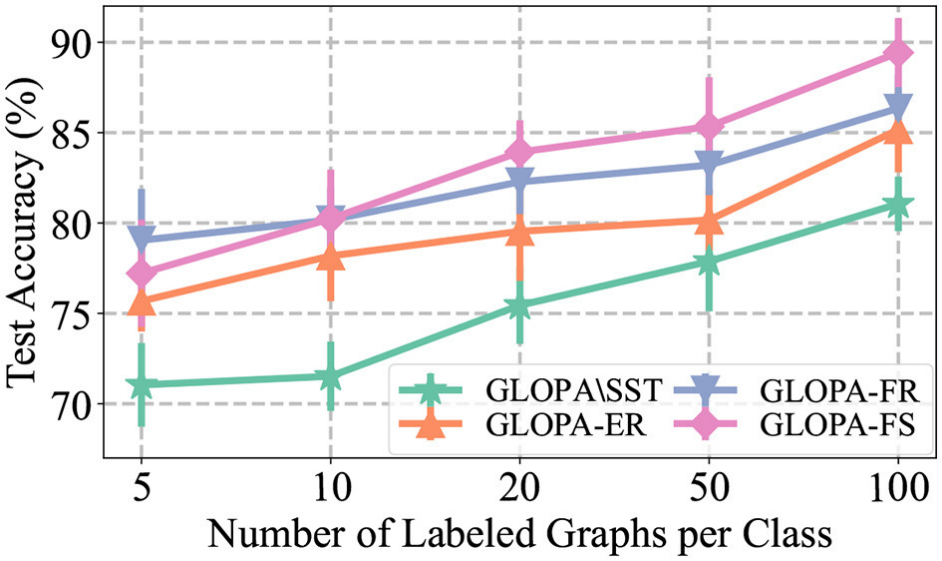
Results under the semi-supervised setting.

To simulate real-world scenarios, where it can be challenging to manually annotate a sufficiently large number of beamline configurations, we conduct experiments with limited labeled data (see Table 1). Specifically, we present the results of using a varying number of labeled beamline graphs per class during SFT, as shown in Figure 4. The following observations can be made from the results: (1) Increasing the amount of labeled data used in the training process leads to a significant improvement in model performance. This improvement is more pronounced for our framework GLOPA without SST, although the absolute performance is still suboptimal due to the absence of SST. (2) When the number of labeled graphs becomes scarce, the performance of all models drops greatly. Nevertheless, the feature removing (FR) strategy becomes more effective in this case. This is due to the fact that by learning from limited node features, the model can better generalize to scenarios with limited labeled beamline graphs for supervised fine-tuning.

### 4.7 Model embedding test

**Figure 5:**
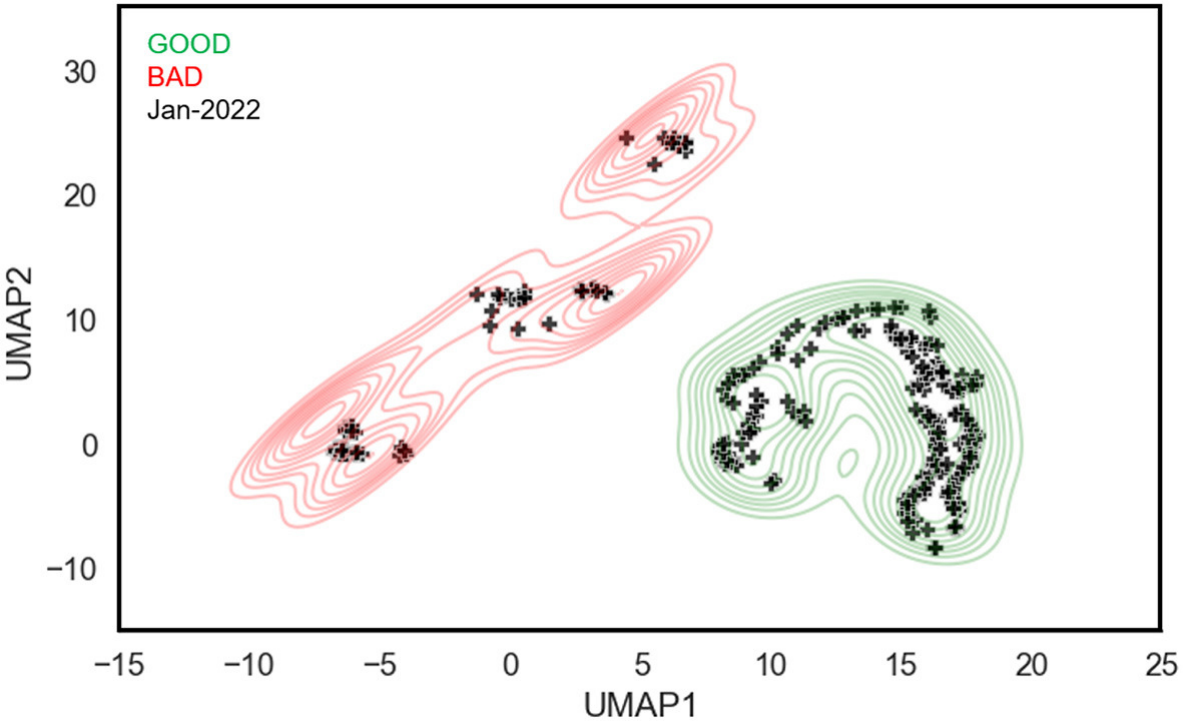
The visualization of 353 unlabeled beamline graphs, representing operations in January 2022 (black markers), using their learned latent embeddings. Green and red contours denote regions of good and bad configurations, respectively.

Following the results of Table 2, we use the GLOPA framework trained with the FS strategy to embed unlabeled graphs in the JAN-2022 dataset, which consists of 353 beamline configurations collected from injector operation during January 2022. To visualize the results, UMAP (McInnes et al., 2018) is used to reduce the dimensionality of the model-generated embeddings, with the results shown in Figure 5. The embeddings from the labeled GOOD and BAD datasets are depicted by the green and red contours, respectively, while the black markers represent the embeddings of the unlabeled data from January 2022. There are several key observations to note from these results: First, the model is able to cleanly separate good and bad regions of parameter space. Second, the majority of the beamline configurations from January 2022 (75%) are clustered in the good region in parameter space, which aligns with our expectation since CEBAF was reliably delivering beam to user end stations during that period. Additionally, because each marker corresponds to a unique timestamp, an expert CEBAF operator is able to look more closely at the beamline configurations in the bad region. Upon investigation, it was confirmed that these configurations were indeed sub-optimal, that is, are appropriately labeled as bad.

## 5 Future work

In this subsection, we provide a comprehensive overview of the future work inspired by our work. The results of Figure 5 motivate further development of this visualization framework to address operational challenges. One application is the ability to track the evolution of a beamline configuration in real time in response to tuning. As noted previously, beam tuning is a time-consuming task that is often driven by trial and error. However, by encoding beamline data into information-rich latent embeddings, operators receive immediate visual feedback about whether their changes are moving the system in the right direction, i.e., toward or away from a region characterized by good configurations. The visualization framework is also uniquely suited to address system stability. The conventional method of monitoring stability requires tracking a set of PVs over time. However, by leveraging low-dimensional embeddings, one can track a many-dimensional space over time. This avoids the need to identify a priori the important PVs and removes the mental burden to operators of monitoring a myriad of signals by eye. Another important avenue of research is to incorporate explainability into the visualization framework. Here the goal is to return to the user a list of the most important nodes that account for, or explain, the difference between two different points in latent space.

## 6 Conclusion

Particle accelerators play a vital role in scientific research. In this work, we have described a new approach to analyze an accelerator beamline by leveraging graph learning to classify good and bad setups. Specifically, our framework represents beamline configurations as heterogeneous graphs and encodes relationships among beamline elements. We then utilize data from both unlabeled and labeled configurations to train a model via our methods of self-supervised training and supervised fine-tuning. Additionally, we demonstrate the ability to leverage a GNN to distill high-dimensional beamline configurations into low-dimensional embeddings and use them to create an intuitive, easy-to-understand visualization for operators. By mapping out regions of latent space characterized by good and bad setups, we describe how this could provide operators with more informative, real-time feedback during beam tuning compared to the standard practice of interpreting a set of sparse, distributed diagnostic readings.

## Data availability statement

The raw data supporting the conclusions of this article will be made available by the authors, without undue reservation.

## Author contributions

SW: Formal Analysis, Methodology, Software, Writing – original draft, Writing – review & editing. CT: Conceptualization, Funding acquisition, Supervision, Visualization, Writing – original draft, Writing – review & editing. DM: Investigation, Writing – review & editing. TL: Data curation, Software, Writing – review & editing. JL: Methodology, Supervision, Writing – review & editing.

## Appendix A

### Discussion of labeled datasets

In this section, we provide additional details on the definition and collection methods for the GOOD and BAD labeled datasets, as introduced in Section 2.1.

### GOOD

A trained CEBAF operator created a set of filters to select periods of stable running representative of what we mean by GOOD. Specifically, these are injector configurations where there had not been a machine trip for 30 minutes, the drive laser modes were set to continuous wave (CW) operation, and the beam current at a diagnostic in the injector exceeded 5 microAmperes. The choice of these filters effectively constrains configurations to those in which the machine was running stably (no machine trips within 30 minutes) and tuned well (able to run moderately high current, CW beam). While the search and filtering can be automated, each event is examined by an experienced operator to confirm that it is indeed “good”.

### BAD

Calling these BAD setups can be somewhat misleading. It would be more correct to think of them as “non-ideal”. Identifying these kinds of setups is very difficult. A configuration in which a beam cannot be cleanly transported through the injector is clearly bad. Yet in those instances, the accelerator's built-in machine protection system (MPS) will shut the machine down to avoid any potential damage. The aim of this work is to identify GOOD setups and be able to distinguish them from non-ideal configurations. To create a set of these latter configurations, we leveraged data from a dedicated beam study. In the study, a variety of beamline components were varied, one at a time, from their nominal values and the downstream response of the system recorded. The changes to each element were sufficient to cause a measurable downstream response, but small enough for the beam to be transported cleanly. These configurations, therefore, reside in that middle ground between GOOD operation and tripping the machine off, which is what we mean by the BAD label in the context of our work.
